# Young Women With Pancreatic Cancer: A High‐Risk Group for Demoralization

**DOI:** 10.1002/pon.70601

**Published:** 2026-09-09

**Authors:** Abrahm Levi, Aryan Nasr, Yujie Cui, Anser A. Abbas, Tara T. Arouchian, Andrew L. Coveler, Tracey L. Pierce, Brian T. Anderson, Eric Collisson, John Davelaar, Simon K. Lo, Srinivas Gaddam, Jun Gong, Arsen Osipov, Andrew E. Hendifar

**Affiliations:** ^1^ Cedars‐Sinai Medical Center Los Angeles California USA; ^2^ Fred Hutchinson Cancer Center Seattle Washington USA; ^3^ University of California San Francisco California USA

**Keywords:** demoralization, demoralization scale 2 (DS‐II), depression, pancreatic cancer, sociodemographic factors

## Abstract

**Background:**

Demoralization is a maladaptive coping response to stressful situations, characterized by thoughts of hopelessness, helplessness, and loss of meaning and purpose. Demoralization is clinically measurable using the Demoralization Scale 2 (DS‐II). Pancreatic cancer (PC) patients, who carry a notoriously poor prognosis, are hypothesized to be at higher risk of demoralization.

**Aims:**

This study uses the DS‐II to gauge the impact of demoralization in PC patients compared to a heterogenous cancer population and establish its relation to depression.

**Methods:**

Patients completed the DS‐II, PHQ‐9, and a demographic survey. The mean DS‐II score of PC patients was compared with that of a previously published heterogenous cancer cohort reported by Ignatius et al. using a one‐sample *t*‐test. The univariable association between DS‐II scores and PHQ‐9 scores was examined using linear regression models.

**Results:**

Of the 206 patients enrolled in the study, 47% were women and the mean age was 67 ± 10 years. The mean DS‐II total score was 4.9 ± 5.2 points, significantly lower than the mean of 10.8 ± 8.0 previously reported by Ignatius and De La Garza II in a psychiatric oncology cohort (*p* < 0.001). There was no significant difference in demoralization scores across disease stages (*p* = 0.8). DS‐II scores in univariable models were associated with depression (*β* = 0.79, *p* < 0.001), age (*β* = −0.09, *p* = 0.008), and women (*p* = 0.002).

**Conclusion:**

Demoralization in this cohort of patients with PC was lower than that reported by Ignatius et al., although differences in recruitment settings and patient characteristics limit direct comparisons. Though disease stage did not impact demoralization scores in PC, our study found demoralization was strongly associated with concurrent depression, young age, and women. Understanding the risk factors associated with demoralization in PC can help enhance the quality of psychosocial care in oncology.

## 1 Background

Demoralization is a maladaptive response to identifiable stressors or existential threats characterized by feelings of helplessness, hopelessness, loss of meaning and purpose, and subjective incompetence (“I can't cope”). Unlike major depression, demoralized individuals retain hedonic capacity and can still experience pleasure, whereas depression is characterized by pervasive anhedonia [1, 2]. Network analyses further support the distinction between these constructs, demonstrating that symptoms of depression and demoralization form largely independent clusters, with low mood and self‐reproach serving as the primary bridges between them [1].

Differentiating demoralization from depression has important clinical implications. First, demoralization carries an independent risk for suicidal ideation. A systematic review of individuals experiencing financial hardship or social isolation found an odds ratio > 6 for suicide risk among those with demoralization, and approximately half of suicidal demoralized individuals did not meet criteria for an affective disorder [3]. Second, misclassifying demoralization as depression may result in unnecessary antidepressant treatment while overlooking interventions that directly address meaning and existential distress. Meaning‐centered psychotherapy and dignity therapy currently have the strongest empirical support for reducing demoralization [4].

The assessment of depression and demoralization also requires distinct screening tools. The Patient Health Questionnaire‐9 (PHQ‐9) is a validated measure of depression but cannot be reliably used to diagnose demoralization [5]. Instead, the Demoralization Scale‐II (DS‐II), a 16‐item self‐reported survey, is used to assess and quantify demoralization [6]. Items are scored from 0 (never) to 2 (often), producing a total score ranging from 0 to 32. Proposed thresholds classify scores of 0–3 as low, 4–10 as moderate, and ≥ 11 as clinically significant demoralization [6].

Pancreatic cancer (PC) is the third leading cause of cancer‐related death in the United States and has a five‐year survival rate of only 13% [7]. Although demoralization has been studied in cancer populations generally, it has not been specifically investigated in PC patients. Given the substantial symptom burden and psychosocial challenges associated with PC, understanding demoralization in this population may help identify unmet supportive care needs and patients at increased risk for suicidality [8, 9, 10].

The primary objective of this study was to compare demoralization in PC patients with previously reported levels in heterogeneous cancer populations. Ignatius and De La Garza II reported a mean DS‐II score of 10.8 (± 8.0) among 922 patients with various cancers [11]. We hypothesized that PC patients would exhibit higher levels of demoralization. Secondary objectives included characterizing average demoralization scores in PC patients and examining their relationship with depression measured by the PHQ‐9. Exploratory objectives assessed associations between demoralization, depression, sociodemographic factors, and disease stage at diagnosis.

## 2 Methods

This prospective, multicenter study evaluated demoralization and depression among patients with PC. Patients were recruited from outpatient oncology clinics at Cedars‐Sinai Medical Center, the University of California San Francisco, and Fred Hutchinson Cancer Center between July 2020 and February 2023 under an IRB‐approved protocol (STUDY00004166). After giving informed consent, patients completed the DS‐II, PHQ‐9, and a self‐reported demographic survey. Patients 18 years of age or older with histologically confirmed or highly suspected pancreatic adenocarcinoma who were able to provide study‐specific informed consent were eligible for this trial. Non‐English‐speaking patients were excluded from this trial as the list of domain questions was not available in other languages. The original protocol also included an exclusion criterion for patients for whom participation was not considered to be in their best interest, in the opinion of the investigator. However, no patient was documented as having been excluded under this criterion during study conduct.

Patient characteristics were summarized as frequencies and proportions for categorical variables and mean (± SD) or median (interquartile range) for continuous variables, as appropriate. Statistical power was calculated using a two‐sided one‐sample *t*‐test comparing DS‐II scores in the study cohort with the published mean score of 10.8 (± 8.0) reported for a heterogeneous cancer population by Ignatius and De La Garza II [11]. A sample size of 200 patients provided 80% power to detect a minimum mean DS‐II difference of 1.6 points.

Associations between DS‐II scores, PHQ‐9 scores, and sociodemographic variables were examined using linear regression models after assessment of model assumptions. Differences in DS‐II scores across disease stages were evaluated using the Kruskal‐Wallis test, while the Jonckheere‐Terpstra test assessed monotonic trends across ordered stages (IA–IV). Linear regression was also used to examine relationships between DS‐II and PHQ‐9 scores and time from diagnosis to survey completion. Statistical significance was defined as *α* = 0.05, and analyses were performed using R version 4.3.3 [12].

To contextualize demographic findings, DS‐II scores were compared with age‐ and gender‐specific normative values reported by Ramm et al. [13] Since the DS‐II has been previously conceptualized as comprising Meaning and Purpose and Distress and Coping Ability domains, confirmatory factor analysis was performed to compare the fit of one‐factor and correlated two‐factor models of demoralization, and internal consistency was assessed using Cronbach's alpha coefficients.

## 3 Results

A total of 206 patients with PC were enrolled between July 2020 and February 2023 across three institutions: Cedars‐Sinai Medical Center (*n* = 167), Fred Hutchinson Cancer Center (*n* = 26), and the University of California, San Francisco (*n* = 13) (Figure S1). The mean age was 67 ± 10 years, and 52% of participants identified as men, 47% as women, and 1% as another gender identity. Most participants were married or partnered (77%). Disease stage at diagnosis ranged from IA to IV, with stage IV disease comprising 40.3% of the cohort (Table 1). Compared with the cohort of patients from Ignatius et al., [11] our sample was older (67.0 vs. 53.3 years), more likely to be male (52% vs. 32%), and more frequently married or partnered (77% vs. 62%). Our population also included a lower proportion of White, non‐Hispanic participants (65% vs. 78%), suggesting a more racially and ethnically diverse cohort.

**TABLE 1:** Patient demographics and characteristics.

Characteristic	*N* = 206^a^
Age	67 ± 10, 68 (61,74)
Stage primary diagnosis	
Stage IA	18 (8.7%)
Stage IB	31 (15%)
Stage IIA	12 (5.8%)
Stage IIB	16 (7.8%)
Stage III	36 (17.5%)
Stage IV	83 (40.3%)
Unknown	10 (4.9%)
Gender identity	
Man	108 (52%)
Man, other gender identity	1 (0.5%)
Other gender identity	1 (0.5%)
Woman	96 (47%)
Race	
American Indian or Alaska native	2 (1.0%)
Another race	8 (3.9%)
Asian	24 (12%)
Black or African American	16 (7.8%)
Middle Eastern or North African	2 (1.0%)
Native Hawaiian or other Pacific Islander	1 (0.5%)
White Hispanic	16 (7.8%)
White non‐Hispanic	133 (65%)
White non‐Hispanic, American Indian or Alaska native	1 (0.5%)
White non‐Hispanic, Middle Eastern or North African	2 (1.0%)
(Missing)	1
Highest education level completed	
No schooling	1 (0.5%)
Nursery school to high school, No diploma	5 (2.4%)
High school graduate or Equivalent (e.g., GED)	19 (9.2%)
Trade/Technical/Vocational training	5 (2.4%)
Some college	29 (14.1%)
2‐Year college degree	25 (12.1%)
4‐Year college degree	60 (29.1%)
Master's degree	31 (15.0%)
Doctoral degree	4 (1.9%)
Professional degree (e.g., M.D., J.D., M.B.A.)	27 (13.1%)
Current occupation	
Homemaker or not working outside the home	17 (8.3%)
Employed (or self‐Employed) full‐time	51 (24.8%)
Employed (or self‐Employe Part‐time	12 (5.8%)
Employed but temporarily away from My job	18 (8.7%)
Unemployed	11 (5.3%)
Retired	95 (46.1%)
(Missing)	2 (1%)
Income	75 (36%)
Under $50,000	50 (24.3%)
$50,000‐$99,998	42 (20.4%)
$99,999‐$150,000	30 (14.5%)
Greater than $150,000	75 (36.4%)
(Missing)	9 (4.4%)
Married or In A relationship	159 (77%)
Religious Affiliation	180 (87%)
Current sexual orientation	
Straight/Heterosexual	195 (94.7%)
Bisexual	2 (1.0%)
Gay	7 (3.3%)
Lesbian	1 (0.5%)
(Missing)	1 (0.5%)
PHQ9 total score	5.5 ± 5.0, 4.0 (2.0, 8.0)
PHQ9 Interpolation total score	
Minimal depression	114 (55.3%)
Mild depression	53 (25.7%)
Moderate depression	27 (13.1%)
Moderately severe depression	9 (4.4%)
Severe depression	3 (1.5%)
DSII total score	4.9 ± 5.2, 4.0 (1.0, 7.0)
(Missing)	1

^a^ Mean ± SD, Median (IQR); *n* (%).

The mean DS‐II total score was 4.9 ± 5.2, significantly lower than the mean score of 10.8 ± 8.0 reported by Ignatius and De La Garza II who surveyed a heterogeneous cancer population in a psychiatric oncology setting (*p* < 0.001) (Table 1). Using previously proposed DS‐II thresholds [6], 101 patients (49.3%) had low demoralization, 79 (38.5%) had moderate demoralization, and 25 (12.2%) had high demoralization (Figure S2a).

The mean PHQ‐9 score was 5.5 ± 5.0, corresponding predominantly to minimal or mild depressive symptoms (Table 1). Demoralization and depression were strongly associated, with each one‐point increase in PHQ‐9 corresponding to a 0.79‐point increase in DS‐II score (*β* = 0.79, *p* < 0.001; Figure S2b).

Mean DS‐II scores did not differ significantly across disease stages (*p* = 0.80) (Table S1), and no monotonic increase in demoralization was observed with advancing stage (Jonckheere‐Terpstra test: JT = 8326.5, *p* = 0.283). Similarly, neither DS‐II nor PHQ‐9 scores were significantly associated with time elapsed since diagnosis (Table S2).

In univariable analyses, higher DS‐II scores were significantly associated with higher PHQ‐9 scores (*β* = 0.79, *p* < 0.001), younger age (*β* = −0.09, *p* = 0.008), and identifying as a woman (*p* = 0.002) (Table 2). For PHQ‐9 scores, only DS‐II score (*β* = 0.74, *p* < 0.001) and younger age (*β* = −0.08, *p* = 0.022) were significant predictors (Table S3).

**TABLE 2:** Risk factors tested against the DS‐II demoralization total score.

Characteristic	N	Beta	95% CI^a^	*p*‐value
PHQ‐9 total score	205	0.79	0.69, 0.88	< 0.001
Age	205	−0.09	−0.16, −0.02	0.008
Gender identity	205			
Man		—	—	
Man, other gender identity		2.2	−7.8, 12	0.7
Other gender identity		5.2	−4.8, 15	0.3
Woman		2.2	0.83, 3.6	0.002
Race	204			
American Indian or Alaska native		—	—	
Another race		3.0	−4.9, 11	0.5
Asian		−0.46	−7.8, 6.9	> 0.9
Black or African American		−1.1	−8.7, 6.4	0.8
Middle Eastern of North African		5.5	−4.5, 16	0.3
Native Hawaiian or other Pacific Islander		−4.5	−17, 7.8	0.5
White Hispanic		−2.3	−9.8, 5.2	0.5
White non‐Hispanic		−2.2	−9.3, 5.0	0.5
White non‐Hispanic, American Indian or Alaska native		−6.5	−19, 5.8	0.3
White non‐Hispanic, Middle Eastern or North African		−0.50	−11, 9.5	> 0.9
Stage primary diagnosis	205			
Stage IA		—	—	
Stage IB		−1.4	−4.5, 1.6	0.4
Stage IIA		−3.3	−7.1, 0.54	0.094
Stage IIB		−0.76	−4.3, 2.8	0.7
Stage III		−1.1	−4.1, 1.8	0.5
Stage IV		−0.93	−3.6, 1.7	0.5
Unknown		−0.84	−4.9, 3.2	0.7
Income	196			
$50,000–$99,998		—	—	
$99,999–$150,000		0.30	−2.2, 2.8	0.8
Greater than $150,000		−0.34	−2.3, 1.6	0.7
Under $50,000		0.56	−1.6, 2.7	0.6
Religion	205			
Not at all		—	—	
Somewhat		2.2	−0.05, 4.4	0.056
Very Much		1.7	−0.57, 4.0	0.14
Occupation	203			
Employed (or self‐employed) full‐time		—	—	
Employed (or self‐employed) part‐time		0.18	−3.1, 3.4	> 0.9
Employed but temporarily away from job		1.6	−1.2, 4.4	0.3
Homemaker or not working outside the home		0.75	−2.1, 3.6	0.6
Retired		−0.71	−2.5, 1.1	0.4
Unemployed		0.46	−2.9, 3.8	0.8
Education	205			
Below college		—	—	
Some college or Above		1.5	−0.50, 3.6	0.14
Occupation	205			
No		—	—	
Yes		0.1	−1.4, 1.6	> 0.9
Income	205			
No		—	—	
Yes		−0.49	−2.0, 0.99	0.5
Relationship status	205			
No		—	—	
Yes		−1.2	−2.9, 0.44	0.15
Religious Affiliation	205			
No		—	—	
Yes		2.0	−0.16, 4.1	0.071
Diagnosis of Metastatic disease	205			
No		—	—	
Yes		0.24	−1.2, 1.7	0.7

^a^ CI = Confidence Interval.

Comparison with age‐ and gender‐specific normative values reported by Ramm et al. [13] demonstrated that participants aged 50–59 years exhibited the highest levels of demoralization relative to population norms. Across all age groups, women reported higher demoralization scores than men, with the greatest difference observed among women aged 50–59 years (Tables [Link], [Link]). Consistent with these findings, gender‐stratified analyses demonstrated higher total DS‐II scores among women than men (6.2 ± 6.0 vs. 3.8 ± 4.2, *p* = 0.001) and higher scores across multiple domains of demoralization, including loss of role (item 3), hopelessness (item 7), emotional sensitivity (item 11), distress regarding their circumstances (item 12), and social isolation (item 15) (Table S5).

Confirmatory factor analysis demonstrated acceptable fit for both one‐factor and two‐factor DS‐II models, with modestly superior fit for the two‐factor model (Table S6). Although all item loadings were significant and internal consistency was high (Cronbach's *α* = 0.902 for the total score, 0.837 for Meaning and Purpose, and 0.833 for Distress and Coping Ability), the correlation between the Meaning and Purpose and Distress and Coping Ability factors was very high (*r* = 0.916), indicating substantial overlap and supporting use of the DS‐II total score as the primary measure of demoralization. Subscale sum scores were 1.7 ± 2.5 and 3.2 ± 3.0 for Meaning and Purpose and Distress and Coping Ability, respectively.

## 4 Discussion

This prospective multi‐center study evaluated demoralization in patients with pancreatic cancer (PC). The mean DS‐II score of 4.9, corresponding to low‐to‐moderate demoralization, was substantially lower than the mean score of 10.8 reported by Ignatius et al. in a heterogeneous cancer population. Notably, Ignatius et al. surveyed patients from psychiatric oncology clinics, where comorbid psychiatric conditions may have contributed to higher demoralization scores [11]. In contrast, Koranyi et al. reported a mean DS‐II score of 5.78 among patients recruited from oncology wards and psychosocial counseling centers [14]. A recent study by Cai et al. found high levels of demoralization (35.45 ± 11.93) among hospitalized PC patients using the original Demoralization Scale (scored 0–96); however, direct comparison is limited by differences in measurement instruments [15].

Overall, our findings indicate that demoralization in this cohort of patients with PC was lower than that reported by Ignatius et al. and similar to that reported by Koranyi et al. However, because these studies differed in recruitment settings and patient characteristics, these comparisons should be interpreted descriptively rather than as evidence that patients with PC experience lower levels of demoralization than the broader oncology population. While the relatively low level of demoralization observed in our cohort may seem paradoxical, given the poor survival rates of PC, emerging evidence suggests that tumor stage and prognostic severity are not robust predictors of demoralization in cancer patients. A systematic review by Hong et al. encompassing 49 studies and 10,712 participants found that advanced tumor stage was associated with demoralization, but the effect size was modest (mean *r* = 0.263) and substantially smaller than those observed for hopelessness (mean r = 0.633), desire for death (mean r = 0.620), dignity‐related distress (mean r = 0.595), depression (mean r = 0.593), anxiety (mean r = 0.589), and physical symptom burden (mean r = 0.331) [16]. Taken together, these findings suggest that demoralization in PC is driven predominantly by psychosocial and symptom‐related factors rather than disease severity alone [16].

Our study found a strong association between demoralization and concurrent depression, young age, and identifying as a woman. However, depression was only correlated with demoralization and young age. Relative to age‐matched normative data from Ramm et al. [13], patients aged 50–59 years demonstrated the greatest elevations in demoralization, while women reported higher scores than men across all age groups, with the largest difference observed among women aged 50–59 years. These findings suggest that younger patients, particularly women, may experience a disproportionate psychosocial burden following a PC diagnosis.

These findings align with existing research suggesting younger cancer patients face greater existential distress and psychological burden, whereas older patients may develop better coping mechanisms and adjusted expectations [17, 18]. Evidence regarding gender differences has been mixed. Tang et al. found no significant association between gender and demoralization in a cohort of Chinese cancer patients, whereas Li et al. identified female gender as an independent predictor of demoralization among elderly patients with advanced lung cancer and recommended targeted interventions for women [19, 20]. In our cohort, identifying as a woman remained a significant risk factor across age groups. This observed difference in gender‐related scores may reflect sociocultural expectations of emotional expression. One study on prostate cancer patients found masculine self‐esteem and resilience were important factors influencing demoralization and suggested men may be less likely to acknowledge demoralization due to concerns about masculinity [21].

### 4.1 Implications

These findings underscore the importance of considering both age and gender when evaluating psychosocial vulnerability in PC. Younger patients demonstrated increased risk for both depression and demoralization, whereas identifying as a woman was associated only with demoralization. Consequently, distinguishing demoralization from depression may be particularly important in younger women with PC. Demoralization is often misdiagnosed as major depression, potentially leading to ineffective antidepressant treatment while overlooking existential concerns that may respond better to meaning‐centered interventions [2, 4, 22].

Although demoralization and depression are strongly correlated, recognizing their distinct characteristics may improve psychosocial care. Given the elevated risk of demoralization among younger women with PC and its established association with suicidality, routine screening using validated measures such as the DS‐II may facilitate earlier identification and targeted intervention [9, 10, 14]. Integrating structured screening with timely mental health support could improve quality of life and reduce suicide risk in this population [23, 24].

### 4.2 Limitations

Strengths of our study include the large, multi‐center sample of patients and the comprehensive exploration of potential risk factors including disease staging and sociodemographic factors such as age, gender, race, income, religion, occupation, education, and relationship status. Limitations of our study include an uneven distribution of patients across sites, no data collected on whether patients received palliative care or other psychosocial or spiritual support that may have “buffered” demoralization, and reliance on comparisons with external historical cohorts that differed in recruitment settings and patient characteristics. These differences may have influenced the observed differences in demoralization scores and limit the ability to draw definitive conclusions from these comparisons.

## 5 Conclusion

Women with PC represent a vulnerable subgroup that are at increased risk of demoralization. This finding has important implications for screening, treatment, and the direction of future research in the field. Current guidelines from the American Society of Clinical Oncology (ASCO) and the American Cancer Society (ACS) recommend routine screening for depression and anxiety, but do not specifically address demoralization [25]. Given our findings that younger women are particularly susceptible to demoralization and that demoralization is associated with increased suicide risk, incorporation of DS‐II screening may help identify patients with unmet psychosocial needs [10]. Clinicians should therefore consider earlier and more proactive psychosocial screening in this subgroup, ensuring that vulnerability is recognized and addressed regardless of disease severity. Embedding psychosocial oncology professionals into routine PC care and tailoring interventions to life milestones could ensure young women receive timely, holistic support addressing their unique psychological needs.

## Funding

This study was supported by internal research funds provided by Dr. Andrew E. Hendifar.

## Ethics Statement

All patients consented to participate on this IRB‐approved study (STUDY00004166).

## Conflicts of Interest

There are no known conflicts of interest to report. Of note, some preliminary data from this manuscript was accepted for a poster presentation at the 2023 American Pancreatic Association conference.

## Supporting information


**Figure S1:** Participant Flow Diagram.

**Figure S2:** Distribution of DS‐II total scores. Scatter Plot of DS‐II and PHQ‐9 Total Scores.

**Table S1:** Demoralization by Disease Stage.

**Table S2:** Linear Regression for DS‐II and PHQ‐9 Total Score Time Lapse.

**Table S3:** Risk Factors Tested Against the PHQ‐9 Depression Score.

**Table S4:** DS‐II Scores By Age Group for PDAC Cohort with Comparison to Age‐Specific Normative Values Reported by Ramm et al. (2023).

**Table S5:** DS‐II Scores By Age Group and Gender for PDAC Cohort with Comparison to Age‐ and Gender‐Specific Normative Values Reported by Ramm et al. (2023).

**Table S6:** Gender‐Stratified Analyses of Total DS‐II and DS‐II Domains.

**Table S7:** Confirmatory Factor Analysis of One‐Factor and Two‐Factor DS‐II Models.

## Data Availability

Any data and analytical methods underlying this article that is not available in the article itself will be shared on reasonable request to the corresponding author.
