# Supplementary material for: Young Women With Pancreatic Cancer: A High‐Risk Group for Demoralization
Source: Psychooncology. 2026 Sep 9;35(9):e70601. doi: 10.1002/pon.70601 (PMC13557214; doi:10.1002/pon.70601)
Supplement: Supplementary file 1 [file PON-35-e70601-s001.pdf]

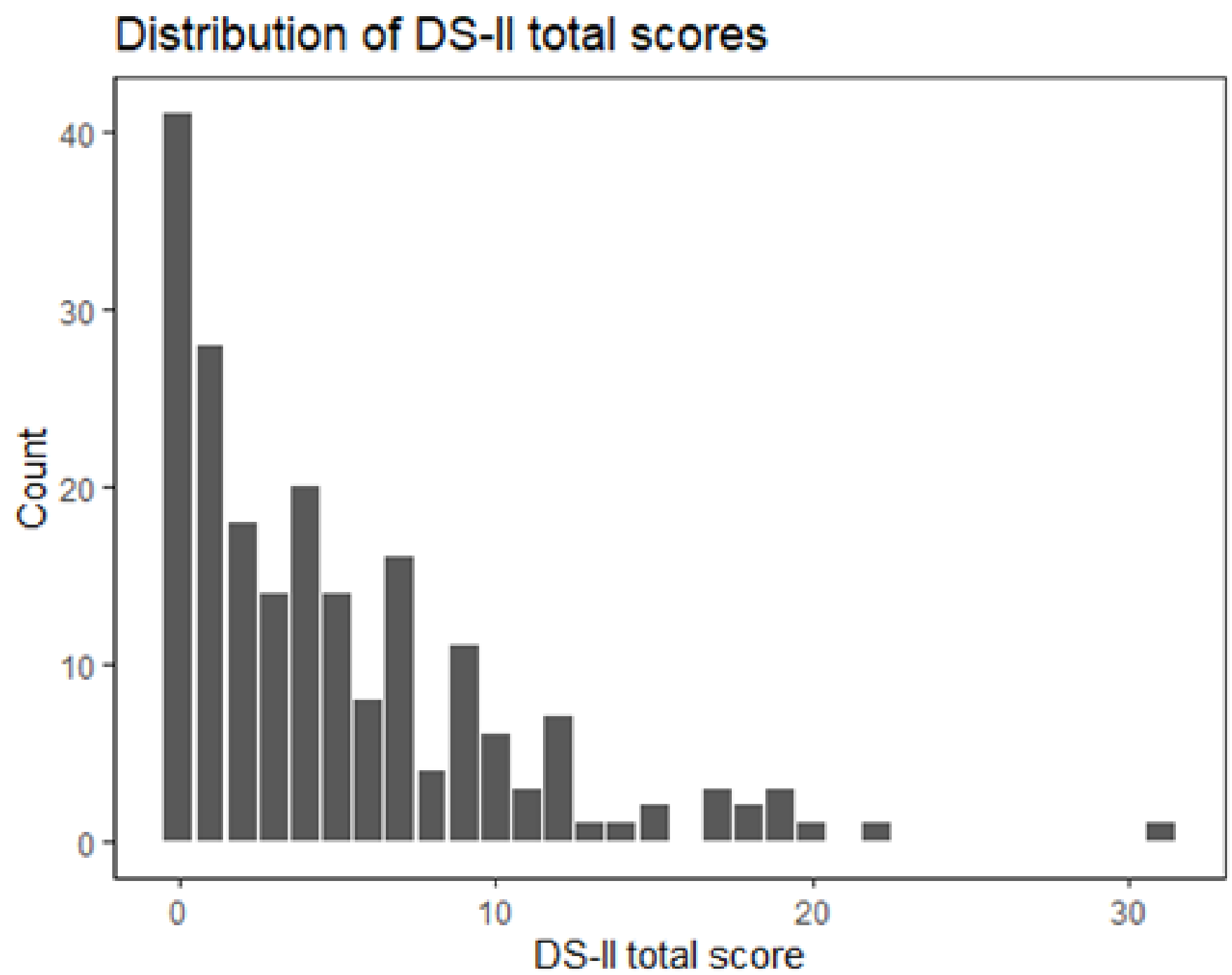

Supplemental Figure 2a. *Distribution of DS-II total scores*

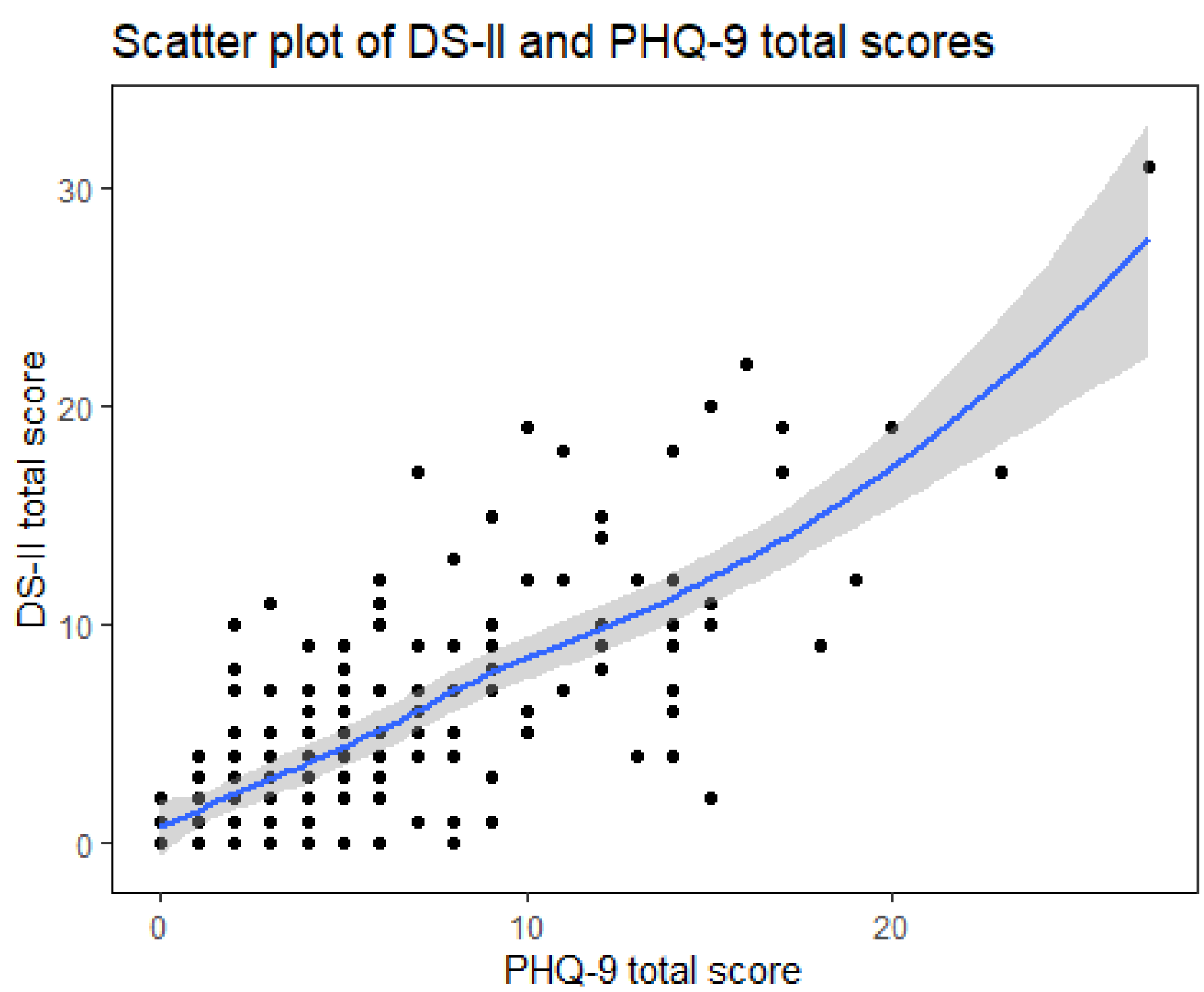

Supplemental Figure 2b. *Scatter Plot of DS-II and PHQ-9 Total Scores*
