# Supplementary material for: Young Women With Pancreatic Cancer: A High‐Risk Group for Demoralization
Source: Psychooncology. 2026 Sep 9;35(9):e70601. doi: 10.1002/pon.70601 (PMC13557214; doi:10.1002/pon.70601)
Supplement: Supplementary file 3 [file PON-35-e70601-s002.docx]

***Supplemental Table 4b:*** *DS-II Scores By Age Group and Gender for PDAC Cohort with Comparison to Age- and Gender-Specific Normative Values Reported by Ramm et al. (2023)*

| **Gender** | **Age Group** | **PDAC N** | **PDAC DS-II Total Mean (SD)** | **Ramm Norm Mean (SD)** | **PDAC - Ramm** | **Interpretation** |
| --- | --- | --- | --- | --- | --- | --- |
| Man | 50-59 | 16 | 4.56 (5.07) | 3.69 (5.35) | 0.87 | Similar to age- and gender-specific norm |
| Man | 60-69 | 38 | 4.61 (4.62) | 3.83 (5.86) | 0.78 | Similar to age- and gender-specific norm |
| Man | >=70 | 45 | 2.87 (3.24) | 3.75 (4.95) | -0.88 | Similar to age- and gender-specific norm |
| Woman | 50-59 | 18 | 9.67 (8.62) | 4.44 (6.25) | 5.23 | Higher than age- and gender-specific norm |
| Woman | 60-69 | 24 | 5.79 (4.75) | 4.57 (6.06) | 1.22 | Similar to age- and gender-specific norm |
| Woman | >=70 | 43 | 5.23 (5.14) | 5.03 (6.25) | 0.20 | Similar to age- and gender-specific norm |
