# Supplementary material for: Young Women With Pancreatic Cancer: A High‐Risk Group for Demoralization
Source: Psychooncology. 2026 Sep 9;35(9):e70601. doi: 10.1002/pon.70601 (PMC13557214; doi:10.1002/pon.70601)
Supplement: Supplementary file 4 [file PON-35-e70601-s003.docx]

***Supplemental Table 4a:*** *DS-II Scores By Age Group for PDAC Cohort with Comparison to Age-Specific Normative Values Reported by Ramm et al. (2023)*

| **Age Group** | **PDAC N** | **PDAC DS-II Total Mean (SD)** | **Ramm Norm Mean (SD)** | **PDAC - Ramm** | **Interpretation** |
| --- | --- | --- | --- | --- | --- |
| 30-39 | 2 | 4.50 (6.36) | 3.27 (5.18) | 1.23 | Similar to age-specific norm |
| 40-49 | 12 | 4.00 (4.26) | 3.20 (5.07) | 0.80 | Similar to age-specific norm |
| 50-59 | 35 | 7.23 (7.42) | 4.07 (5.83) | 3.16 | Higher than age-specific norm |
| 60-69 | 62 | 5.06 (4.67) | 4.19 (5.97) | 0.87 | Similar to age-specific norm |
| >=70 | 89 | 4.08 (4.42) | 4.40 (5.67) | -0.32 | Similar to age-specific norm |

Ramm reference values are from Supplement Table S2 of Ramm et al. (2023).

Interpretation compares the Ramm norm mean with the 95% confidence interval of the PDAC mean within each age group.
