# Supplementary material for: Young Women With Pancreatic Cancer: A High‐Risk Group for Demoralization
Source: Psychooncology. 2026 Sep 9;35(9):e70601. doi: 10.1002/pon.70601 (PMC13557214; doi:10.1002/pon.70601)
Supplement: Supplementary file 5 [file PON-35-e70601-s005.docx]

***Supplemental Table 2:*** *Linear Regression for DS-II and PHQ-9 Total Score Time Lapse*

| Term |  | β coefficient | Error | Statistic | p-value |
| --- | --- | --- | --- | --- | --- |
| Time Lapse |  |  |  |  |  |
|  | DS-II total score | -0.001 | 0.001 | -1.536 | 0.126 |
|  | PHQ-9 total score | -0.001 | 0.001 | -1.943 | 0.053 |
