# Supplementary material for: Young Women With Pancreatic Cancer: A High‐Risk Group for Demoralization
Source: Psychooncology. 2026 Sep 9;35(9):e70601. doi: 10.1002/pon.70601 (PMC13557214; doi:10.1002/pon.70601)
Supplement: Supplementary file 6 [file PON-35-e70601-s006.docx]

***Supplemental Table 1:*** *Demoralization by Disease Stage*

| Characteristic |  | Stage IA,  N= 18^1^ | Stage IB, N=31^1^ | Stage IIA, N=12^1^ | Stage IIB, N=16^1^ | Stage III, N= 36^1^ | Stage IV, N=83^1^ | Unknown, N= 10^1^ | p-value^2^ |
| --- | --- | --- | --- | --- | --- | --- | --- | --- | --- |
| DSII Total Score |  | 5.9 ± 7.3,  3.5 (1.0, 8.8) | 4.5 ± 5.0,  3.0 (0.0, 5.0) | 2.7 ± 3.0,  1.0 (0.0, 5.0) | 5.2 ± 5.3,  4.5 (0.8, 7.5) | 4.8 ± 5.1,  3.5 (1.0, 7.0) | 5.0 ± 5.1,  4.0 (1.0, 7.0) | 5.1 ± 4.6,  3.5 (2.0, 6.5) | 0.8 |

^1^Mean ± SD,

Median (IQR)

^2^Kruskal-Wallis rank sum test
