# Supplementary material for: Young Women With Pancreatic Cancer: A High‐Risk Group for Demoralization
Source: Psychooncology. 2026 Sep 9;35(9):e70601. doi: 10.1002/pon.70601 (PMC13557214; doi:10.1002/pon.70601)
Supplement: Supplementary file 7 [file PON-35-e70601-s007.docx]

***Supplementary Table 5:*** *Gender-Stratified Analyses of Total DS-II and DS-II Domains*

| **Characteristic** | **Man (N=108^1^)** | **Woman (N=96^1^)** | **p-value^2^** |
| --- | --- | --- | --- |
| DS-II Item 1: There is little value in what I can offer others. |  |  | 0.3 |
| 0 | 86 (80%) | 67 (71%) |  |
| 1 | 19 (18%) | 22 (23%) |  |
| 2 | 3 (2.8%) | 6 (6.3%) |  |
| (Missing) | 0 | 1 |  |
| DS-II Item 2: My life seems to be pointless |  |  | 0.073 |
| 0 | 99 (93%) | 78 (82%) |  |
| 1 | 7 (6.5%) | 14 (15%) |  |
| 2 | 1 (0.9%) | 3 (3.2%) |  |
| (Missing) | 1 | 1 |  |
| DS-II Item 3: My role in life has been lost. |  |  | **0.023** |
| 0 | 86 (80%) | 66 (69%) |  |
| 1 | 21 (19%) | 21 (22%) |  |
| 2 | 1 (0.9%) | 8 (8.4%) |  |
| (Missing) | 0 | 1 |  |
| DS-II Item 4: I no longer feel emotionally in control. |  |  | 0.070 |
| 0 | 85 (79%) | 61 (65%) |  |
| 1 | 21 (19%) | 28 (30%) |  |
| 2 | 2 (1.9%) | 5 (5.3%) |  |
| (Missing) | 0 | 2 |  |
| DS-II Item 5: No one can help me. |  |  | 0.3 |
| 0 | 90 (83%) | 76 (81%) |  |
| 1 | 15 (14%) | 18 (19%) |  |
| 2 | 3 (2.8%) | 0 (0%) |  |
| (Missing) | 0 | 2 |  |
| DS-II Item 6: I feel that I cannot help myself |  |  | 0.6 |
| 0 | 81 (75%) | 66 (69%) |  |
| 1 | 24 (22%) | 24 (25%) |  |
| 2 | 3 (2.8%) | 5 (5.3%) |  |
| (Missing) | 0 | 1 |  |
| DS-II Item 7: I feel hopeless. |  |  | **0.003** |
| 0 | 88 (81%) | 59 (62%) |  |
| 1 | 19 (18%) | 30 (32%) |  |
| 2 | 1 (0.9%) | 6 (6.3%) |  |
| (Missing) | 0 | 1 |  |
| DS-II Item 8: I feel irritable. |  |  | 0.4 |
| 0 | 57 (53%) | 42 (44%) |  |
| 1 | 46 (43%) | 50 (53%) |  |
| 2 | 5 (4.6%) | 3 (3.2%) |  |
| (Missing) | 0 | 1 |  |
| DS-II Item 9: I do not cope well with life. |  |  | 0.2 |
| 0 | 95 (88%) | 77 (81%) |  |
| 1 | 13 (12%) | 16 (17%) |  |
| 2 | 0 (0%) | 2 (2.1%) |  |
| (Missing) | 0 | 1 |  |
| DS-II Item 10: I have a lot of regret about my life. |  |  | 0.2 |
| 0 | 87 (81%) | 68 (72%) |  |
| 1 | 20 (19%) | 24 (25%) |  |
| 2 | 1 (0.9%) | 3 (3.2%) |  |
| (Missing) | 0 | 1 |  |
| DS-II Item 11: I tend to feel hurt easily. |  |  | **0.002** |
| 0 | 91 (84%) | 62 (66%) |  |
| 1 | 17 (16%) | 28 (30%) |  |
| 2 | 0 (0%) | 4 (4.3%) |  |
| (Missing) | 0 | 2 |  |
| DS-II Item 12: I feel distressed about what is happening to me. |  |  | **0.002** |
| 0 | 45 (42%) | 19 (20%) |  |
| 1 | 48 (44%) | 51 (54%) |  |
| 2 | 15 (14%) | 25 (26%) |  |
| (Missing) | 0 | 1 |  |
| DS-II Item 13: I am not a worthwhile person. |  |  | 0.15 |
| 0 | 105 (97%) | 87 (92%) |  |
| 1 | 3 (2.8%) | 7 (7.4%) |  |
| 2 | 0 (0%) | 1 (1.1%) |  |
| (Missing) | 0 | 1 |  |
| DS-II Item 14: I would rather not be alive. |  |  | 0.3 |
| 0 | 103 (95%) | 87 (92%) |  |
| 1 | 5 (4.6%) | 6 (6.3%) |  |
| 2 | 0 (0%) | 2 (2.1%) |  |
| (Missing) | 0 | 1 |  |
| DS-II Item 15: I feel quite isolated or alone. |  |  | **0.015** |
| 0 | 97 (90%) | 70 (74%) |  |
| 1 | 9 (8.3%) | 19 (20%) |  |
| 2 | 2 (1.9%) | 5 (5.3%) |  |
| (Missing) | 0 | 2 |  |
| DS-II Item 16: I feel trapped by what is happening to me. |  |  | 0.2 |
| 0 | 68 (64%) | 49 (52%) |  |
| 1 | 29 (27%) | 31 (33%) |  |
| 2 | 10 (9.3%) | 15 (16%) |  |
| (Missing) | 1 | 1 |  |
| DS-II total score | 3.8 ± 4.2,  3.0 (0.0, 5.0) | 6.2 ± 6.0,  5.0 (1.0, 9.0) | **0.001** |
| (Missing) | 2 | 4 |  |

^1^n (%); Mean ± SD,

Median (Q1, Q3)

^2^Fisher's exact test; Pearson's Chi-squared test; Wilcoxon rank sum test
