# Supplementary material for: Young Women With Pancreatic Cancer: A High‐Risk Group for Demoralization
Source: Psychooncology. 2026 Sep 9;35(9):e70601. doi: 10.1002/pon.70601 (PMC13557214; doi:10.1002/pon.70601)
Supplement: Supplementary file 8 [file PON-35-e70601-s008.docx]

***Supplemental Table 3:*** *Risk Factors Tested Against the PHQ-9 Depression Score*

| **Characteristic** |  | **N** | **Beta** | **95%CI^1^** | **p-value** |
| --- | --- | --- | --- | --- | --- |
| DS-II Total Score |  | 205 | 0.74 | 0.65, 0.82 | <0.001 |
| Age |  | 206 | -0.08 | -0.14, -0.01 | 0.022 |
| Gender Identity |  | 206 |  |  |  |
|  | Man |  | – | – |  |
|  | Man, Other Gender Identity |  | 5.0 | -4.8, 15 | 0.3 |
|  | Other Gender Identity |  | 4.0 | -5.8, 14 | 0.4 |
|  | Woman |  | 0.98 | -0.40, 2.4 | 0.2 |
| Race |  | 205 |  |  |  |
|  | American Indian or Alaska Native |  | – | – |  |
|  | Another race |  | -2.5 | -10, 5.2 | 0.5 |
|  | Asian |  | -4.6 | -12, 2.6 | 0.2 |
|  | Black or African American |  | -5.1 | -12, 2.6 | 0.2 |
|  | Middle Eastern of North African |  | 0.50 | -9.3, 10 | >0.9 |
|  | Native Hawaiian or other Pacific Islander |  | -6.0 | -18, 6.0 | 0.3 |
|  | White Hispanic |  | -5.4 | -13, 1.9 | 0.15 |
|  | White non-Hispanic |  | -6.1 | -13, 0.86 | 0.088 |
|  | White non-Hispanic, American Indian or Alaska Native |  | -11 | -23, 0.95 | 0.073 |
|  | White non-Hispanic, Middle Eastern or North African |  | -3.0 | -13, 6.8 | 0.5 |
| Stage Primary Diagnosis |  | 206 |  |  |  |
|  | Stage IA |  | – | – |  |
|  | Stage IB |  | -0.40 | -3.3, 2.5 | 0.8 |
|  | Stage IIA |  | -2.1 | -5.8, 1.6 | 0.3 |
|  | Stage IIB |  | -0.16 | -3.6, 3.2 | >0.9 |
|  | Stage III |  | -0.58 | -3.4, 2.3 | 0.7 |
|  | Stage IV |  | 0.12 | -2.5, 2.7 | >0.9 |
|  | Unknown |  | -0.02 | -3.9, 3.9 | >0.9 |
| Income |  | 197 |  |  |  |
|  | $50,000 - $99,998 |  | – | – |  |
|  | $99,999 - $150,000 |  | -0.47 | -2.8, 1.9 | 0.7 |
|  | Greater than $150,000 |  | 0.24 | -1.7, 2.2 | 0.8 |
|  | Under $50,000 |  | 1.4 | -0.70, 3.5 | 0.2 |
| Religion |  | 206 |  |  |  |
|  | Not at all |  | – | – |  |
|  | Somewhat |  | 1.2 | -0.98, 3.4 | 0.3 |
|  | Very Much |  | 0.91 | -1.3, 3.1 | 0.4 |
| Occupation |  | 204 |  |  |  |
|  | Employed (or self-employed) full-time |  | – | – |  |
|  | Employed (or self-employed) part-time |  | 0.17 | -3.0, 3.3 | >0.9 |
|  | Employed but temporarily away from job |  | 1.1 | -1.6, 3.8 | 0.4 |
|  | Homemaker or not working outside the home |  | 1.1 | -1.6, 3.9 | 0.4 |
|  | Retired |  | -0.42 | -2.1, 1.3 | 0.6 |
|  | Unemployed |  | 0.41 | -2.9, 3.7 | 0.8 |
| Education |  | 206 |  |  |  |
|  | Below College |  | – | – |  |
|  | Some College or Above |  | 0.20 | -1.7, 2.1 | 0.8 |
| Occupation |  | 206 |  |  |  |
|  | No |  | – | – |  |
|  | Yes |  | -0.04 | -1.5, 1.5 | >0.9 |
| Income |  | 206 |  |  |  |
|  | No |  | – | – |  |
|  | Yes |  | 0.01 | -1.4, 1.4 | >0.9 |
| Relationship Status |  | 206 |  |  |  |
|  | No |  | – | – |  |
|  | Yes |  | -1.3 | -2.9, 0.31 | 0.11 |
| Religious Affiliation |  | 206 |  |  |  |
|  | No |  | – | – |  |
|  | Yes |  | 1.1 | -0.99, 3.1 | 0.3 |
| Diagnosis of Metastatic Disease |  | 206 |  |  |  |
|  | No |  | – | – |  |
|  | Yes |  | 0.62 | -0.77, 2.0 | 0.4 |

^1^CI = Confidence Interval
