# Supplementary material for: Young Women With Pancreatic Cancer: A High‐Risk Group for Demoralization
Source: Psychooncology. 2026 Sep 9;35(9):e70601. doi: 10.1002/pon.70601 (PMC13557214; doi:10.1002/pon.70601)
Supplement: Supplementary file 9 [file PON-35-e70601-s009.docx]

***Supplementary Table 6:*** *Confirmatory Factor Analysis of One-Factor and Two-Factor DS-II Models*

| **Model** | **X^2^ (df)** | **CFI** | **TLI** | **RM SEA (90% CI)** | **SRM R** | **SABIC** |
| --- | --- | --- | --- | --- | --- | --- |
| 1-factor model | 286.0 (104) | 0.806 | 0.776 | 0.092 (0.082; 0.103) | 0.075 | 3,416 |
| 2-factor model | 271.8 (103) | 0.820 | 0.790 | 0.089 (0.079; 0.100) | 0.074 | 3,394 |

X^2^ = chi-square test; df = degrees of freedom; CFI = Comparative Fit Index; TLI = Tucker-Lewis Index; RMSEA = Root Mean Square Error of Approximation; SRMR = Standardized Root Mean Squared Residual; CI = confidence interval; SABIC = sample-size adjusted Bayesian information criterion.

This table is based on the MLR sensitivity analysis to mirror the presentation used by Ramm et al (2023).
